# Semiembedded HDPE/VIS Composite Membranes with Tailored Wettability and Superior Mechanical Durability for Outdoor Protective Applications

**DOI:** 10.1021/acsomega.5c03862

**Published:** 2025-07-29

**Authors:** Heng Zhang, Qian Zhai, Qi Zhen, Hongbin Lv, Kongmeng Ye

**Affiliations:** † College of intelligent textile and fabric electronics, 118217 Zhongyuan University of Technology, No. 1 Huaihe Road, Xinzheng, Zhengzhou, Henan 451191, P.R. China; ‡ College of fashion technology, 118217 Zhongyuan University of Technology, No. 1 Huaihe Road, Xinzheng, Zhengzhou, Henan 451191, P.R. China; § ZFJ Textile machinery Co, LTD., No.258 Wutong Street, Zhengzhou, Henan 450000, P.R. China; ∥ Kingwills Advanced Materials Co, Ltd., No.166 Jianghai Road, Chongchuan, Nantong, Jiangsu 226017, P.R. China

## Abstract

Outdoor personal protective equipment (O-PPE) is vital for health and safety during activities, such as climbing and camping. However, achieving comfort in using O-PPE is challenging owing to subpar liquid management and rigid textures. In this study, a semiembedded HDPE/VIS microfibrous membrane was developed by hydroentangling a high-density polyethylene (HDPE) microfibrous web fabricated via flash spinning with a viscose (VIS) fibrous web created through carding. This innovative structure was formed under the impact of water jets, where HDPE microfibers were embedded in the VIS fiber layer, resulting in a dense and stable composite structure. This semiembedded structure enables the rapid wettability of the VIS layer while maintaining the hydrophobicity of the HDPE layer, thereby significantly enhancing liquid management, as demonstrated by a high accumulative one-way transport index of 1036%. Additionally, the sample exhibits a high water-vapor transmission rate of 1973 g/(m^2^ ·24 h), and the HDPE layer effectively obstructs air. Furthermore, the membrane exhibits a promising daytime radiative cooling property, thus resulting in a lower maximum temperature by 12.9 °C, a superior softness of 0.24 N, and uniform mechanical performance, which can be enhanced by adjusting the water-jet pressure and HDPE mass per unit area. Overall, the HDPE/VIS microfibrous membrane provides a balanced combination of protection, comfort, and durability, thereby offering a versatile solution for advanced O-PPE.

## Graphical abstract

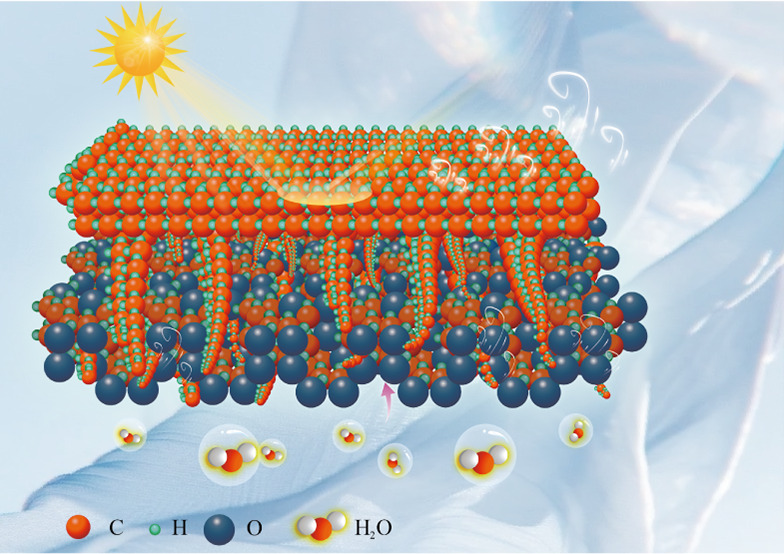

## 1 Introduction

Personal protective equipment (PPE) is crucial in ensuring the health and safety of individuals in hazardous environments.^1^ Recently, the significance of comfort in using PPE has gained prominence, as outdoor pursuits such as sports, camping, and travel have increasingly become essential components of a satisfying lifestyle.^2^ High-density polyethylene (HDPE) microfibrous membranes^3^ are known for their favorable mechanical properties, large surface area, dense pores, excellent weather resistance, and maintenance of stable performance under various adverse climatic conditions.^4^ Consequently, they are widely used in outdoor personal protective equipment (O-PPE). However, pure HDPE microfibrous membranes^5^ exhibit disadvantages such as subpar liquid management and a plastic feel devoid of a soft touch, thus rendering them ineffective in personal thermal-moisture management, particularly during intense activities or in hot environments.^6^ Therefore, HDPE microfibrous membranes that provide excellent liquid management ability and favorable tactile comfort need to be developed for O-PPE applications.

Recently, several techniques such as electrospinning,^7^ melt blowing,^8^ and flash spinning^9^ have been attempted to fabricate HDPE microfibrous membranes. Among them, flash spinning^10^ presents significant advantages for creating HDPE microfibrous membranes, including high productivity, uniform microfiber distribution, and fine pore structure. However, HDPE microfibrous membranes^11^ fabricated via flash spinning are uncomfortable for users. Currently, researchers are developing improved fibrous membranes. Liu et al.^12^ prepared a novel breathable and waterproof composite fabric with a three-layer structure using a thermal pressing process. After laminating the thermoplastic polyurethane (TPU) membrane, the breathability remained as high as 13,956 g/m^2^ ·d, maintaining both protective properties and comfort. Zhou et al.^13^ developed a fibrous membrane filtration material by constructing a gradient wetting structure comprising a hydrophobic outer layer, a dust-filtration middle layer, and a moisture-permeable inner layer. This design not only enhances the performance of individual protective materials but also provides excellent comfort. Thus, these structured fibrous membranes^14^ can effectively preserve material functionality while enhancing comfort, offering a more balanced solution for advanced material applications.

Hydroentanglement technology^15^ is a green preparation process involving the application of high-pressure water jets to promote fiber entanglement, which consolidates the fibers into a stable structure and avoids the use of chemical adhesives.^16^ Moreover, hydroentanglement effectively incorporates various anisotropic fibrous webs to achieve multiple functions. For example, Lu et al.^17^ developed a comfortable nonwoven spunlace fabric by hydroentangling polyimide and lyocell fibers, which offered rapid liquid penetration and excellent antibacterial properties. This chemical-free process is a crucial prerequisite for the practical application of materials in the hygiene field. Sun et al.^18^ used high-speed water jets to disperse moisture-absorbing viscose fibers onto hydrophobic polylactic acid fibers, thereby forming discontinuous, hydrophobic, and partially hydrophilic layers with directionally hydrophobic properties. The development of such structures highlights the versatility and effectiveness of hydroentanglement in the creation of advanced materials with enhanced functionalities.

In this study, an HDPE/VIS microfibrous membrane was hydroentangled with HDPE microfibrous webs obtained via flash spinning and viscose fibrous webs prepared via carding into an integrated microfibrous membrane by using high-pressure water jets. During hydroentanglement, the HDPE microfibers were embedded into the viscose fibrous web, forming a semiembedded structure. This semiembedded microfibrous structure not only enhanced the liquid management capacity but also improved the softness performance, thus providing an option for multifunctional O-PPE applications that combine protection and comfort.

## 2 Experimental Section

### 2.1 Materials

HDPE pellets (5000S), −C_2_H_4_–, with a density of 0.95–0.96 g/cm^3^, and a melt flow index of 0.3–0.5 g/10 min (190 °C, 2.16 kg), were purchased from Beijing Yanshan Petrochemical Co., Ltd. The viscose fibers, −C_6_H_10_O_5_–, with a fineness of 1.56 dtex and a length of 38 mm, were purchased from Tangshan Sanyou Group Co., Ltd. (Hebei, China). The dichloromethane and methanol were kindly provided by Kingwills Advanced Materials Co., Ltd. (Jiangsu, China). The main specifications of the HDPE pellets and viscose fibers are shown in Tables S1 andS2.

### 2.2 Preparation

#### 2.2.1 Preparation of HDPE Microfibrous Webs by Flash Spinning Process

Figure 1a illustrates the flash-spinning process used to prepare HDPE microfibrous webs. The HDPE pellets were heated and melted by a screw extruder at 200 °C. Subsequently, the HDPE melt was dissolved into an HDPE solution with a mass ratio of 16.5% using a mixture of dichloromethane and methanol with a mass ratio of 80:20 in a kettle at 200 °C and 15,000 kPa. The HDPE solution was then sprayed into the atmosphere through a decompression chamber, where the solvent mixture instantly evaporated to form HDPE microfibers. These microfibers were separated, redirected using deflector plates, and deposited onto a belt to form an HDPE microfibrous web. The masses per unit area of the prepared HDPE microfibrous webs (*M*_*HDPEfs*_) were 37.1, 56.8, 65.7, 81.9, and 94.2 g/m^2^.

**Figure 1:**
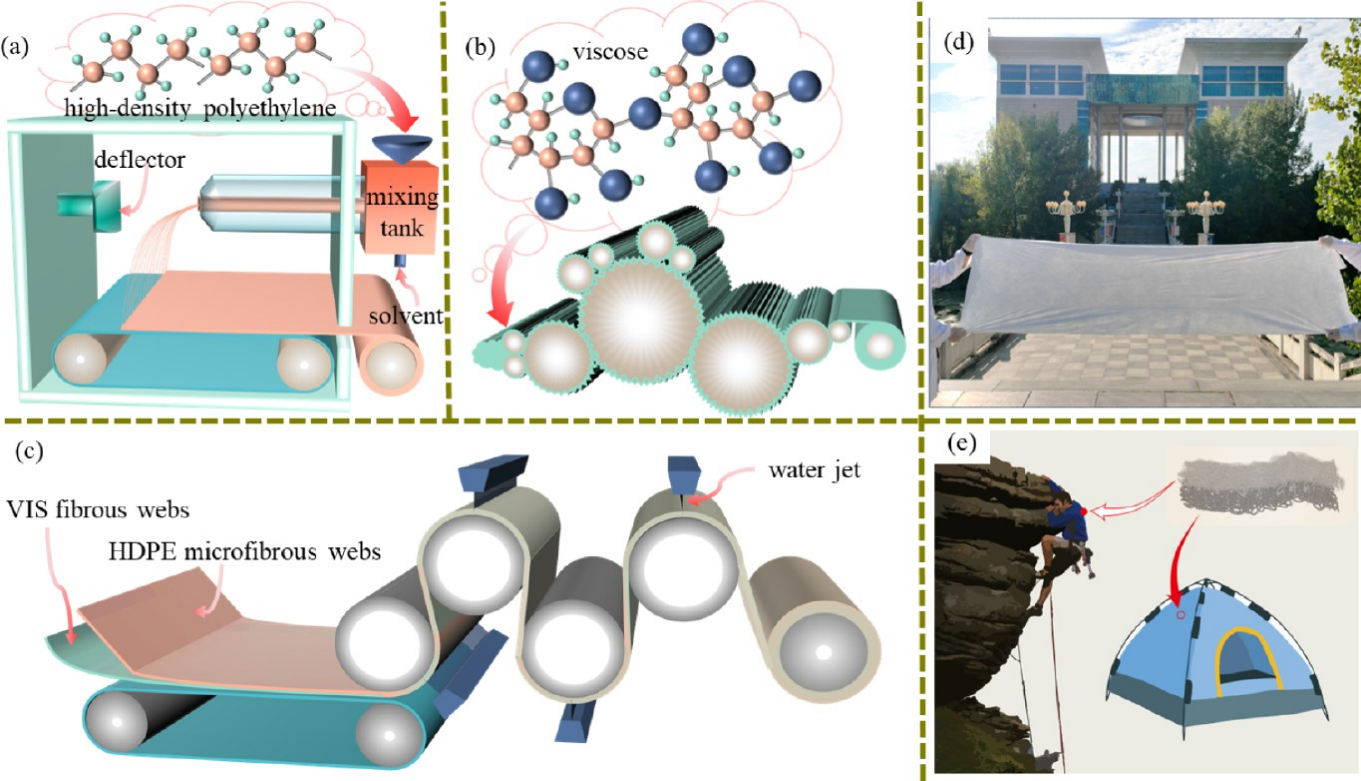
Schematic diagram showing the preparation of HDPE microfibrous webs, viscose fibrous webs, and HDPE/VIS microfibrous membranes via (**a**) flash spinning, (**b**) carding, and (**c**) hydroentangling. Photograph showing (**d**) large, light, flat, and thin prepared HDPE/VIS microfibrous membrane samples and (**e**) their versatile O-PPE applications, such as climbing, skiing, and camping.

#### 2.2.2 Preparation of Viscose Fibrous Webs by Carding Process

The viscose fibers were opened and fed into a roller carding system (Card-WL-600, Changshu Wanlong Machinery Co., Ltd., China), comprising a cylinder, three workers, and three strippers. Under the action of these components (Figure 1b), the fibers overlapped to form a viscose fibrous web. Subsequently, this web was fed into a cross-laying system (CL-WL-600, Changshu Wanlong Machinery Co., Ltd., China) and lapped into webs with a density of 59.6 g/m^2^.

#### 2.2.3 Preparation of the HDPE/VIS Microfibrous Membrane by Hydroentanglement Process

The viscose fibrous webs and HDPE microfibrous webs were further laminated and fed into a hydroentangling tester (Figure 1c, SC600, HI-TECH Heavy Industry Co., Ltd., China). The tester comprised one prewet injector and four main injectors. Water pressures (*Pw*) of 70, 90, 110, 130, and 150 bar were applied to bond the viscose fibrous webs and HDPE microfibrous webs, while maintaining a consistent speed of 3 m/min. The main hydroentanglement parameters are listed in Table S3. Additionally, Figure 1d shows that the fabricated HDPE/VIS microfibrous membrane is large yet light, flat, and thin, demonstrating its potential for O-PPE applications in climbing, skipping, and camping (Figure 1e).

### 2.3 Characterization

The surface and cross-sectional morphologies of the samples were characterized by using scanning electron microscopy (SEM; JSM-IT 200, JEOL Ltd., Japan). Prior to scanning, the samples were gold-coated and observed at an accelerating voltage of 10 kV. The fiber diameter distribution was obtained by using the free software Smile View Map (JEOL, Japan). The pore-size distributions of the samples were determined by using a pore-size analyzer (PSM 165H, Topas, Germany). The thickness and porosity were tested in accordance with GB/T 24218.1 and GB/T 24218.2. The liquid contact angles were measured by using a digital goniometer (SDC350, Dongguan Shengding Precision Instrument Co., Ltd.). The wicking speeds of the liquids on both sides of each sample were measured by using a custom-developed device. The liquid moisture management ability was measured by using a moisture-management tester with 0.9% NaCl solution as the test liquid. Air permeability was tested by using an air permeability tester (YG461E; Wenzhou Darong Textile Instrument, China). The water-vapor transmission rates (*WVTR*s) of the samples were measured using a water-vapor transmission-rate tester (W3/031; Jinan Languang, China). Water-vapor transmission was further tested by using a custom-developed device. The tensile breaking strength, elongation at break, Young’s modulus, yield stress, bursting strength, and tearing strength of the samples were tested using an electronic fabric strength tester (HD026S, Nantong Hongda Experimental Instrument Co., Ltd.) in accordance with GB/T 24218.3 and GB/T3917.3. The reflectivity of the samples was measured by using a UV–visible spectrophotometer (Color i5; X-rite, USA). The infrared spectrum curve and emissivity of the samples were tested by using a Fourier transform infrared spectrometer (Nicolet 6700, Thermo Fisher, USA) equipped with an integrating sphere accessory. The radiative cooling abilities of the samples were tested using a custom-made radiative cooling device comprising a foam box wrapped in aluminum foil and a multichannel temperature tester. The solar irradiance was measured using a solar power meter (TES1333R, TES, Taiwan, China). Real-time temperature changes of the model were measured by using an infrared thermal camera (P20Max; HIKMICRO, China). The softness was evaluated using the custom-developed “Handle-Ometer” testing system based on ASTM D 2923-95.

## 3 Results and Discussion

### 3.1 Semiembedding Structure of HDPE/VIS Microfibrous Membrane

The structure of a microfibrous membrane significantly affects its performance.^19^ Therefore, the semiembedded structure of the HDPE/VIS microfibrous membrane was characterized using SEM, along with its structural parameters such as pore size and porosity. As shown in Figure 2a,b, the semiembedded microfibrous membrane structure comprised an HDPE layer and a VIS layer. The VIS layer (Figure 2c) was highly porosity, which facilitated the penetration of air and water vapor. By contrast, the HDPE layer (Figure 2d) exhibited a dense network morphology, which provided high strength, waterproof properties, and sunlight reflection. HDPE microfibers with a uniform diameter distribution of 4–6 μm (Figure 2e) were partially embedded into the VIS layer in the form of HDPE microfibrous clusters under the impact of high-pressure water jets, creating a semiembedded structure. The SEM images confirmed that the HDPE microfibers successfully penetrated and embedded into the VIS layer during the hydroentanglement process, whereas the VIS fibers did not penetrate the HDPE layer. This resulted in a robust semiembedded structure where the HDPE fibers contributed to the mechanical strength and the VIS fibers enhanced the breathability and moisture management. These HDPE microfibrous clusters were formed when the HDPE microfibers were integrated with the viscose fibers, establishing a strong connection at the interface between the HDPE and VIS layers. The VIS layer contained intertwined HDPE microfibers; however, no VIS fibers were observed in the HDPE layer. This suggests that coarser and more flexible VIS fibers cannot penetrate the denser HDPE layer under the same hydrodynamic conditions, thus highlighting the directional nature of the embedding process. High-pressure water jets penetrated the HDPE layer, transporting the HDPE microfibers and anchoring them within the coarser viscose fibers. The interlayer physical entanglement enhanced the practicality of the HDPE/VIS microfibrous membrane and improved its mechanical strength and durability. The design and performance of the semiembedded HDPE/VIS microfibrous membrane were primarily determined by two key factors: the thickness (mass per unit area) of the HDPE layer and the water-jet pressure during the hydroentanglement process.^20^

**Figure 2:**
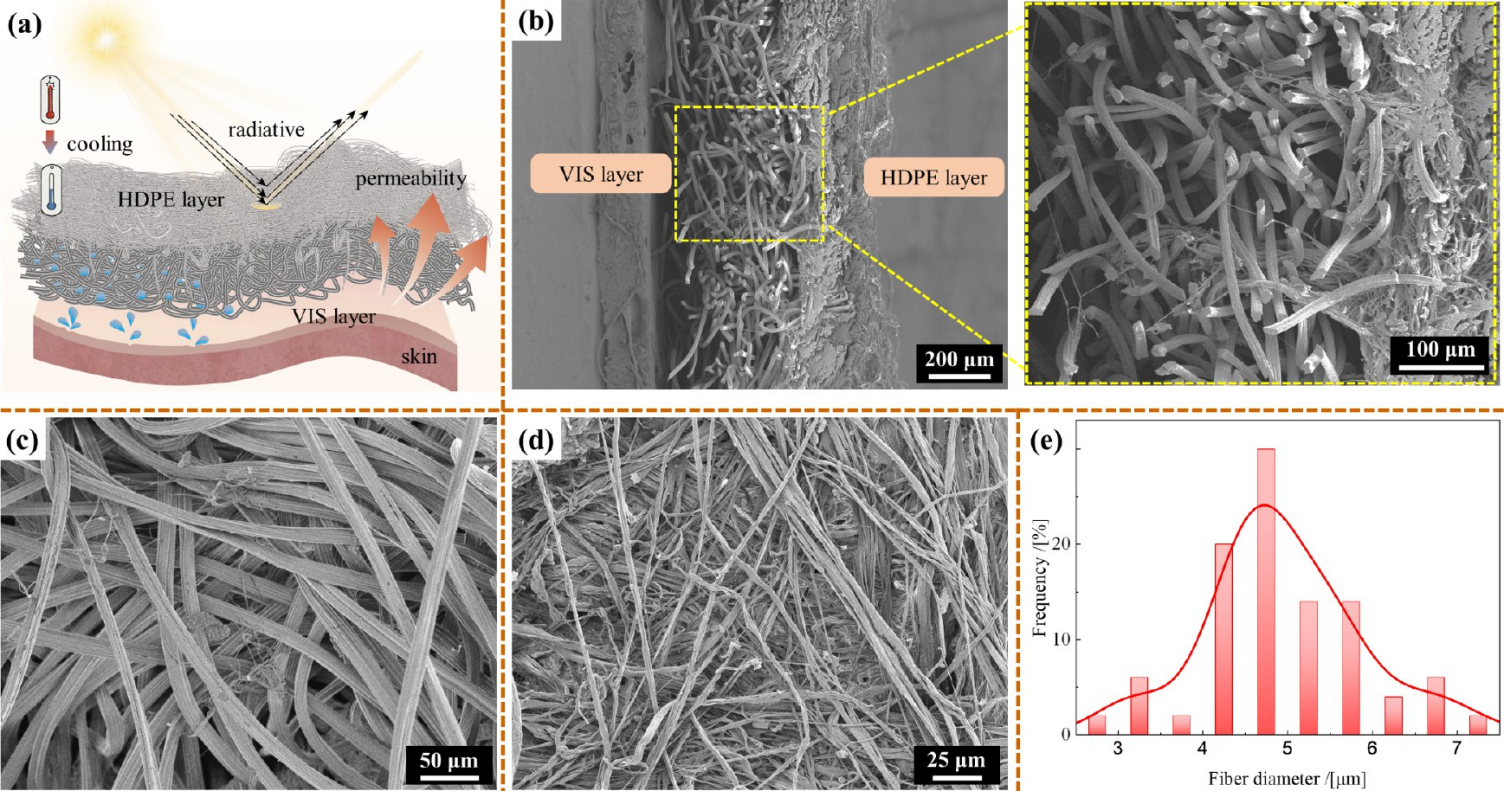
(**a**) Schematic diagram showing the semiembedding structure of HDPE/VIS microfibrous membrane. SEM images of HDPE/VIS microfibrous membrane samples with *M*_*HDPEfs*_ of 56.8 g/m^2^ prepared at *Pw* = 110 bar, as viewed from the (**b**) cross-section, (**c**) VIS layer, and (**d**) HDPE layer. (**e**) Fiber diameter distribution of HDPE microfibrous webs.

Figure 3 presents the SEM images of the HDPE layer and the cross-sections of the HDPE/VIS microfibrous membranes prepared at different water-jet pressures. The HDPE layer exhibited a nonwoven structure with round microfibers randomly distributed horizontally. Additionally, unlike conventional flash-spun nonwovens, the HDPE layer of this sample exhibited the typical hydroentangled nonwoven structural characteristics, where the microfibers were entangled with each other, resulting in “fibrous bundling” on the surface. Comparing Figure 3a–c, when *Pw* was 90 bar, the HDPE layer showed a relatively loose microfibrous network with visible gaps and less fiber entanglement. As *Pw* increased to 130 bar, the HDPE layer became denser with better fiber interlocking and reduced visible gaps. Further increasing *Pw* to 150 bar resulted in tightly packed HDPE microfibers with significant interlocking and minimal gaps, which resembled a highly compact structure. Simultaneously, Figure 3d–f shows that when *Pw* was less than 90 bar, the semiembedded structure was not prominent, indicating that the HDPE microfibers were not embedded deeply into the VIS layer. However, when *Pw* exceeded 130 bar, the HDPE microfibers became more deeply integrated into the VIS layer. Thus, a higher *Pw* results in a denser semiembedded structure owing to enhanced fiber interlocking and embedding; this is confirmed by the pore-size distribution. Additionally, the “fibrous bundling” becomes more pronounced as *Pw* increases. Figure 3g,h shows that increasing *Pw* from 70 to 150 bar decreased the modal pore size, thickness, and porosity of the prepared samples. For the HDPE/VIS microfibrous membrane sample with *M*_*HDPEfs*_ = 56.8 g/m^2^, when *Pw* was 70 bar, its thickness, porosity, and modal pore size were 0.677 mm, 88.1%, and 19.6 μm, respectively. When *Pw* increased to 150 bar, its thickness, porosity, and modal pore size decreased to 0.539 mm, 84.1%, and 7.9 μm, respectively. This occurred because the high-pressure water jets enhanced the displacement of the fibers, resulting in a denser entanglement and a decrease in the volume and size of the interfiber pores. Additionally, the narrower pore-size distribution confirmed that the prepared samples became more uniform and tighter as *Pw* increased. The modal pore size was inversely proportional to *M*_*HDPEfs*_ (Figure 3i). As *M*_HDPEfs_ increased from 37.1 to 94.2 g/m^2^, the number of stacked fiber layers in the thickness direction of the sample increased, causing the modal pore size to decrease from 19.6 to 6.7 μm. Notably, the asymmetric bilayer structure of the HDPE/VIS microfibrous membrane and its semiembedded structure, which were formed by interwoven fiber clusters, resulted in asymmetric wettability. This structure^21^ enhanced the complementary functions of the VIS and HDPE layers. Consequently, the rapid, one-way expulsion of body fluids from the skin was achievable, allowing for dry and comfortable skin.

**Figure 3:**
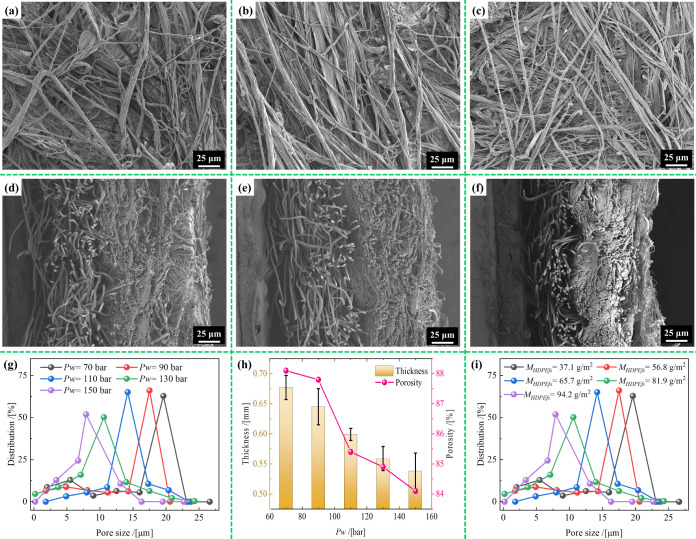
HDPE layer and cross-section SEM images of HDPE/VIS microfibrous membrane samples with *M*_*HDPEfs*_ = 56.8 g/m^2^ prepared at *Pw* of (**a**,**d**) 90, (**b**,**e**) 130, and (**c**,**f**) 150 bar. Physical structural characteristics. (**g**) Pore-size distribution, (**h**) thickness, and porosity of samples prepared at different water pressures. (**i**) Pore-size distribution of samples with different *M*_*HDPEfs*_ prepared at *Pw* = 110 bar.

### 3.2 Liquid-Management Capabilities

Figure 4 illustrates the liquid-wetting performance of HDPE microfibrous webs and VIS fibrous webs. Figure 4a shows the interaction between a water droplet and the VIS fibrous webs. Upon contact, the water droplet dispersed rapidly, indicating that the VIS fibrous webs can rapidly absorb and efficiently channel liquid.^22^ This property is crucial for the inner layer of the O-PPE, as it facilitates the rapid wicking of sweat from the skin, thereby managing moisture and maintaining a dry microclimate, which prevents discomfort and skin irritation during extended wear. By contrast, Figure 4b shows the high hydrophobicity of the HDPE microfibrous webs. When the water droplet initially established contact with the webs (*t* = 0 s), it maintained a near-spherical shape with a contact angle of 143°. This angle remained unchanged for 10 s, indicating that the water droplets did not disperse on the surface and highlighting the liquid-repellent ability of the HDPE layer. Figure 4c shows the performance of various liquid droplets, i.e., water, cola, milk, and coffee, on the HDPE microfibrous webs. Despite their different surface tensions, all droplets maintained circular shapes, confirming the effectiveness of the HDPE layer as a liquid barrier.^23^ Figure S1 displays the contact angles of water, cola, milk, and coffee on the HDPE microfibrous webs, which were 145°, 148°, 144°, and 149°, respectively. This further demonstrates the excellent liquid barrier performance of the prepared HDPE microfibrous web against various liquids. This broad-spectrum hydrophobic performance is particularly beneficial for O-PPE used in diverse outdoor conditions, as the HDPE microfibrous webs prevent rain or other liquids from penetrating, ensuring comfort and dryness. The wicking properties of the HDPE/VIS microfibrous membrane samples were further analyzed (shown in Figure 4d,e1,e2). The wicking–time curve for the VIS layer increased sharply initially and reached a substantial wicking height within 20 s. By contrast, the wicking–time curve for the HDPE layer remained relatively flat, with a minimal change in wicking height over time. This stark difference in the slopes of the two curves highlights the significant difference in wetting properties between the layers, emphasizing the dual functionality of the HDPE/VIS microfibrous membrane. The HDPE layer provides external liquid protection, whereas the VIS layer ensures moisture management and comfort, maintaining membrane durability and user comfort under various environmental conditions.^24^

**Figure 4:**
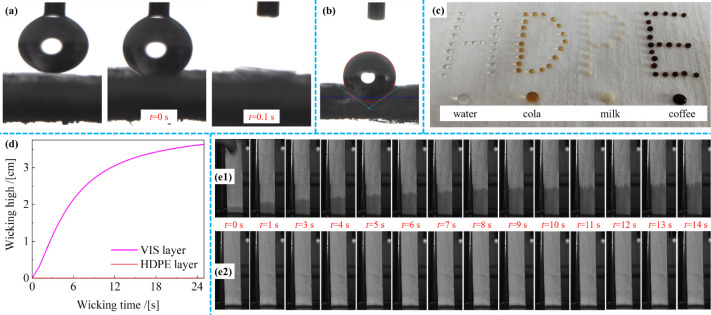
Water contact angle testing results of (**a**) VIS fibrous webs and (**b**) HDPE microfibrous webs. (**d**) Liquid droplets of water, cola, milk, and coffee placed on HDPE microfibrous webs. (**d**) Wicking height–time curves and (**e1-**VIS layer, **e2**-HDPE layer) profiles of HDPE/VIS microfibrous membrane samples with *M*_*HDPEfs*_ = 56.8 g/m^2^ prepared at *Pw* = 110 bar.

Physiological saline was applied to both the VIS and HDPE layers to evaluate the effect of the semiembedded structure of the HDPE/VIS microfibrous membrane. The results of the accumulative one-way transport index (*AOTI*) and the corresponding water content–time curves are presented in Figure 5a–f. When liquid water was applied to the HDPE layer, the *AOTI* increased from 656% to 1036% as the *M*_*HDPEfs*_ decreased from 94.2 to 37.1 g/m^2^ (Figure 5a). For the sample with *M* *_HDPEfs_* = 37.1 g/m^2^ (Figure 5b), the water content in the VIS layer reached 1205% in 24.5 s. By contrast, for the sample with *M*_*HDPEfs*_ = 81.9 g/m^2^ (Figure 5c), the water content in the VIS layer reached 1000% within 22.5 s. These results indicate that increasing *M*_HDPEfs_ decreased the liquid transport capacity. In other words, the thicker of the HDPE layer, the more difficult it is for liquid water to traverse from the HDPE layer (top) to the VIS layer (bottom).^25^ Figure 5d illustrates the variation in *AOTI* for samples with *M*_*HDPEfs*_ = 56.8 g/m^2^ when liquid water was applied to the VIS layer. As *Pw* increased from 70 to 150 bar, *AOTI* changed from −1377% to −1203%. For the samples prepared at 90 bar (Figure 5e), the water content in the VIS layer rapidly increased to approximately 1484% within 22.9 s, whereas that in the HDPE layer remained at approximately 86%. By contrast, for the sample prepared at 130 bar (Figure 5f), the water content in the VIS layer reached approximately 1557% within 22.1 s, whereas that in the HDPE layer increased to approximately 35%. These findings indicate that increasing *Pw* enhanced fiber embedding, resulting in a more compact structure that impeded water transport from the VIS layer (top) to the HDPE layer (bottom). Consequently, the semiembedded structure of the HDPE/VIS microfibrous membrane functions as an effective unidirectional liquid-transmission system, comprising an HDPE layer that prevents liquid penetration and a VIS layer that absorbs and diffuses moisture (Figure 5g).^26^ When water droplets were applied to the HDPE layer (Figure 5h1, Movie S1), they did not diffuse over 160 s; but vertically infiltrated the VIS layer, diffusing rapidly within it. By contrast, droplets contacting the VIS layer (Figure 5h2, Movie S2) rapidly wetted it without penetrating the HDPE layer. These results demonstrate the excellent liquid-management performance of the semiembedded HDPE/VIS microfibrous membrane, ensuring both comfort and durability. The *AOTI* can be easily regulated by adjusting the *M*_*HDPEfs*_ and *Pw*, enabling the optimization of the liquid-transport capabilities.

**Figure 5:**
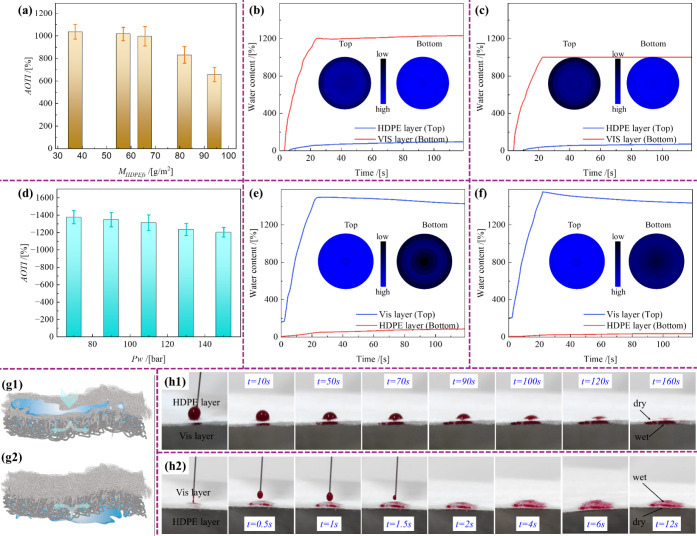
Liquid water pumped on the HDPE layer at 120 s; the (**a**) *AOTI* varying with *M*_*HDPEfs*_; and water content–time curves of HDPE/VIS microfibrous membrane samples with *M*_*HDPEfs*_ of (**b**) 37.1 and (**c**) 81.9 g/m^2^ prepared at *Pw* = 110 bar. Water pumped on the VIS layer at 120 s; (**d**) *AOTI* varying with *Pw*; water content–time curves of HDPE/VIS microfibrous membrane samples with *M*_*HDPEfs*_ of 56.8 g/m^2^ prepared at *Pw* of (**e**) 90 and (**f**) 130 bar. (**g1**,**g2**) Schematic diagrams showing liquid one-way transport and (**h1**,**h2**) liquid wetting behavior for water pumped on HDPE and VIS layers, respectively.

### 3.3 Breathability Assessment

The breathability assessment of the HDPE/VIS microfibrous membranes is crucial for ensuring both the comfort and functionality of the O-PPE. To evaluate this aspect, the air permeability and *WVTR* were assessed using experimental methods and numerical simulations. As illustrated in Figure 6a, the air permeability of the membranes decreased from 39.46 to 22.15 mm/s as *M*_*HDPEfs*_ increased from 37.1 to 94.2 g/m^2^. This reduction is attributed to HDPE microfiber densification, resulting in smaller pore size distribution. Consequently, airflow penetration becomes increasingly restricted as air navigates through a more complex fiber network. In Figure 6b, the air permeability of the samples with *M*_*HDPEfs*_ = 56.8 g/m^2^ is depicted at various *Pw*. At *Pw* values of 70, 90, 110, 130, and 150 bar, the air permeability values were 44.6, 37.9, 31.5, 30.6, and 27.9 mm/s, respectively. The decrease with increasing *Pw* is due to the enhanced fiber entanglement and densification of the HDPE layers, reducing the space available for airflow. Numerical simulations were conducted to investigate the effectiveness of the semiembedded structure of the HDPE/VIS microfibrous membranes in outdoor applications. The virtual structure, pressure contour, and pressure-layer curves (Figure 6c) provide a detailed visualization of the airflow pressure distribution. The HDPE microfibers (blue) and viscose fibers (yellow) were interwoven to form a multilayer fibrous network. This dense network resulted in reduced air permeability and increased airflow resistance.^27^ The pressure contours indicate higher-pressure concentrations in the HDPE layer. The pressure-layer curves further confirm that airflow resistance occurred primarily within the HDPE layer, significantly impeding the airflow. These results demonstrate that the HDPE layer effectively obstructed wind, preserving warmth and comfort under harsh weather conditions. These findings are consistent with the experimental observations and suggest that balancing breathability and wind resistance requires optimization of the HDPE microfiber layer structure by adjusting *M* *_HDPEfs_* and *Pw*.

**Figure 6:**
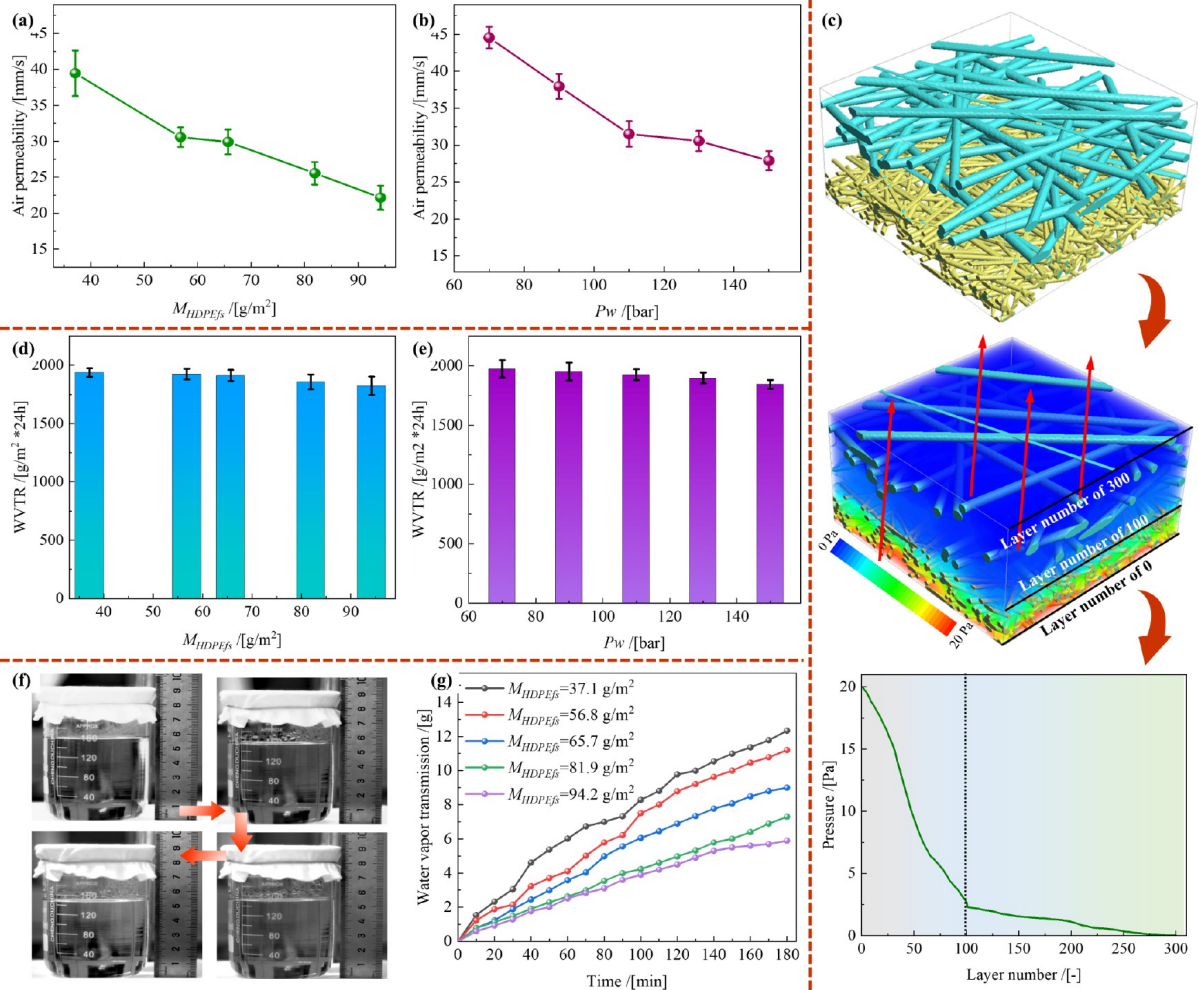
Air permeability of prepared HDPE/VIS microfibrous membrane samples varying with (**a**) *M*_*HDPEfs*_ and (**b**) *Pw*. (**c**) Virtual structures, pressure contour, and pressure-layer curves of the air-permeability simulation. *WVTR* of prepared HDPE/VIS microfibrous membrane samples varying with (**d**) *M*_*HDPEfs*_ and (**e**) *Pw*. (**f**) Self-designed water-vapor-transmission device and (**g**) water vapor transmission–time curves of HDPE/VIS microfibrous membrane samples with different *M*_*HDPEfs*_ values prepared at *Pw* of 110 bar.

The *WVTR* is a key factor in maintaining a dry and comfortable microenvironment within the O-PPE. As expected, the *WVTR* of the samples decreased slightly as both *M* *_HDPEfs_* (Figure 6d) and *Pw* (Figure 6e) increased. Specifically, at *Pw* values of 70, 90, 110, 130, and 150 bar, the *WVTR*s were 1973.2, 1950.0, 1923.9, 1896.8, and 1842.7 g/(m^2^ ·24 h), respectively. Additionally, the *WVTR* decreased from 1936.6 to 1823.7 g/(m^2^ ·24 h) as *M*_*HDPEfs*_ increased from 37.1 to 94.2 g/m^2^. This trend aligns with the air-permeability findings, i.e., thinner and looser HDPE/VIS microfibrous membranes encourage the movement of water vapor.^28^ A custom-designed water-vapor-transmission device (Figure 6f) was utilized to simulate the *WVTR* in hot, humid (45 °C) and high-activity conditions. The water vapor transmission–time curves (Figure 6g) indicate that the water vapor transmission increased linearly with the testing time, thus confirming the high *WVTR* values of the prepared HDPE/VIS microfibrous membrane samples. For the samples with *M* *_HDPEfs_* values of 37.1, 56.8, 65.7, 81.9, and 94.2 g/m^2^, the cumulative water vapor permeation values were 12.3, 11.2, 9.0, 7.3, and 5.9 g, respectively. In conclusion, the semiembedded HDPE/VIS microfibrous membranes comprising both VIS and HDPE layers demonstrated windproof and breathable properties, rapidly transferring moisture outward to facilitate air/water vapor penetration, regulate body temperature, and maintain dryness. These properties render them suitable for advanced O-PPE applications.^29^

### 3.4 Mechanical Properties

The mechanical properties are critical for O-PPE applications^30^ and are determined significantly by the *M*_HDPEfs_.^31^ Tensile strength,^32^ Young’s modulus,^33^ yield stress,^34^ tearing strength, and bursting strength were tested and analyzed to comprehensively evaluate the effect of *M*_HDPEfs_ on the mechanical performance.^35^ The results are shown in Figures 7 and S2, and are summarized in Table S4. The results provide insights into the behavior of HDPE/VIS microfibrous membranes under various forms of mechanical stress. The tensile force–displacement and stress–strain curves (Figures 7a,b and S2) indicate that with the increase of *M*_HDPEfs_, the tensile breaking force, Young’s modulus, and yield stress all increased accordingly. Specifically, in the cross direction (CD), the tensile breaking forces for the samples with *M*_*HDPEfs*_ values of 37.1, 56.8, 65.7, 81.9, and 94.2 g/m^2^ were 126.5, 149.6, 183.2, 206.2, and 217.7 N/5 cm. In the machine direction (MD), the corresponding values were 139.9, 159.6, 211.8, 220.3, and 249.6 N/5 cm. The tensile breaking elongation decreased slightly from 77.4% to 57.3% in the CD and from 58.2% to 36.9% in the MD with increasing *M*_*HDPEfs*_. This indicates that higher *M*_*HDPEfs*_ values enhance the resistance to deformation under external forces. Similarly, the Young’s modulus and yield stress in the CD increased from 8.3 and 3.8 to 17.8 and 6.6 MPa, representing increases of 114.5% and 73.7%, respectively. In the MD, these values increased from 12.8 and 4.2 to 23.6 and 7.6 MPa, representing increases of 84.4% and 81.0%, respectively. These results indicate that the stiffness of the samples also increased with increasing *M*_HDPEfs_. This phenomenon can be attributed to the increased number of fibers per unit area with higher *M*_*HDPEfs*_, which enhances the interfiber bonding strength. Meanwhile, the bursting force–displacement curves (Figure 7c) revealed a significant increase in the bursting breaking force from 187.4 to 364.1 N as *M*_HDPEfs_ increased from 37.1 to 94.2 g/m^2^. This suggests that the samples became less deformable under pressure, beneficial for maintaining structural integrity in high-stress applications. The nonlinear curves indicate that hydroentangled HDPE/VIS microfibrous membranes possess a loose microstructure, making them susceptible to deformation and elongation under external force. As stretching continued, the fibers aligned along the force direction and became taut, accompanied by breaking and slipping of some fibers. In the later stages of stretching, as the number of fiber breaks and slips increased, the microfibrous membrane underwent tensile fracture, causing the force to decline significantly from its maximum value. Moreover, the tearing force–displacement curves in MD and CD (Figure 7d,e, respectively) show similar trends. The tearing breaking forces increased from 52.8 to 104.7 N in the CD and from 66.6 to 127.4 N in the MD with increasing *M*_HDPEfs_. The curves suggest that the samples with higher *M*_HDPEfs_ exhibited less deformation at the peak forces, indicating greater stiffness and durability, attributed to higher fiber density and enhanced interfiber interactions. Figure 7f1,f2 show the tearing behavior of the HDPE/VIS microfibrous membranes in both the CD and MD, and the corresponding videos are shown in Movie S3 and Movie S4, respectively. Under the applied tearing force, the deformations were relatively uniform in both directions. The fibers around the stress points gradually disintegrated, and the tears propagated progressively. However, the tears were not concentrated at a single point; instead, they occurred along a longer path, thus indicating that the tearing force was distributed over a larger area. This longer tear path suggests that the HDPE/VIS microfibrous membranes effectively resisted the tearing forces in both the CD and MD. Additionally, the observed improvements in the tensile, bursting, and tearing properties with increasing *M*_*HDPEfs*_ are attributable to the following reasons: 1) A higher *M* *_HDPEfs_* results in a denser microstructure, enhancing interfiber bonding and structural integrity. 2) An increased fiber density results in a better load distribution across the membrane, thus reducing localized stress concentrations.^36^ In summary, HDPE/VIS microfibrous membranes with higher *M*_*HDPEfs*_ exhibit superior, uniform mechanical properties, rendering them suitable for applications requiring high strength and durability. These findings highlight the potential of these membranes for O-PPE and other mechanically demanding applications.

**Figure 7:**
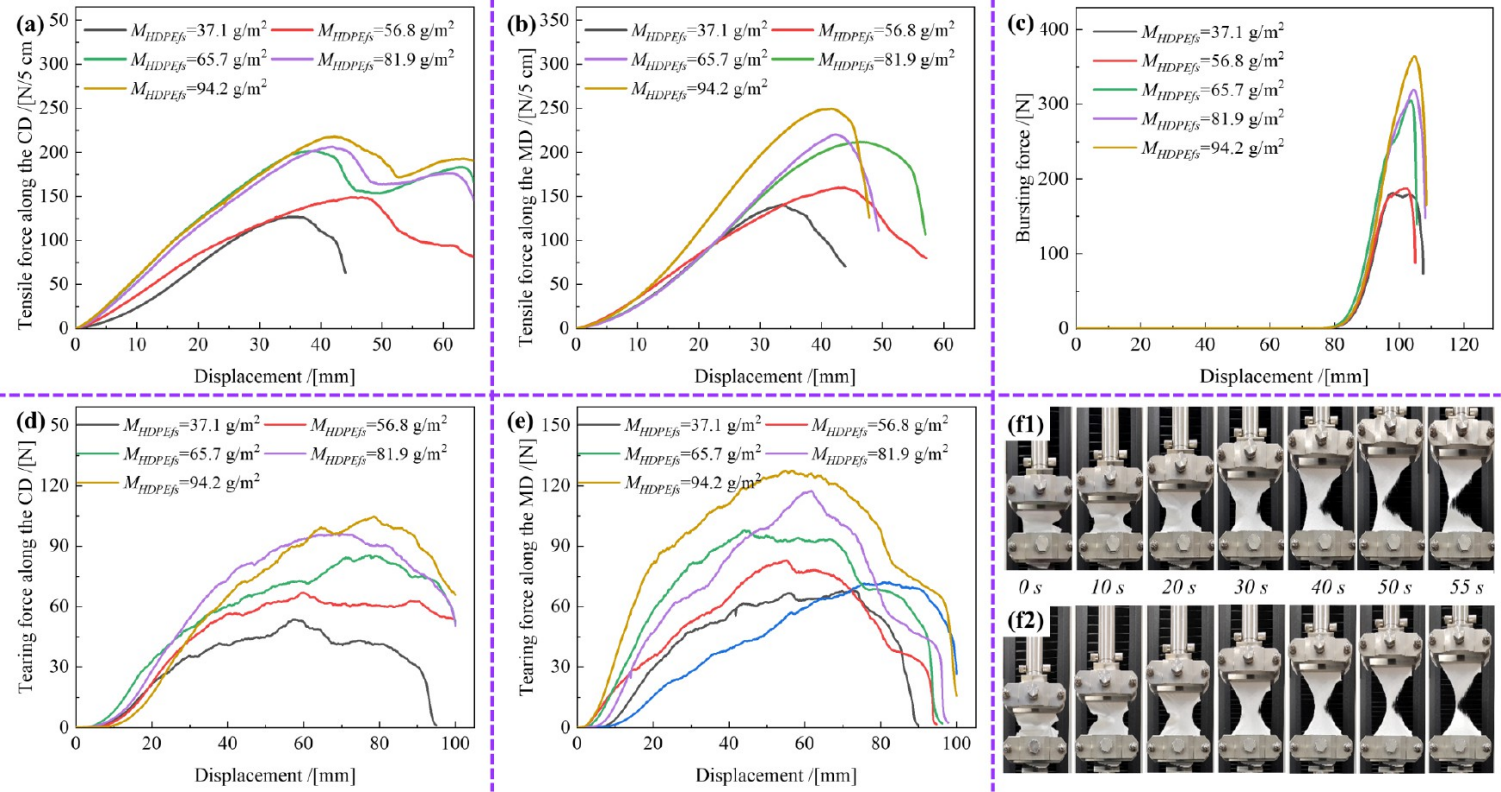
Tensile force–displacement curves (**a**-CD, **b**-MD) and (**c**) bursting force–displacement and tearing force–displacement curves (**d**-CD, **e**-MD) of HDPE/VIS microfibrous membrane samples with different *M*_*HDPEfs*_ prepared at *Pw* = 110 bar. Photograph of the tearing process along (**f1)** CD and (**f2**) MD of HDPE/VIS microfibrous membrane samples with *M*_*HDPEfs*_ of 56.8 g/m^2^ prepared at *Pw* = 110 bar.

### 3.5 Daytime Radiative Cooling Properties

Figure 8 shows the reflectivity and the daytime radiative cooling performance of the HDPE/VIS microfibrous membrane samples. And the emissivity curve of the sample prepared at *Pw* = 130 bar is shown in Figure S3. Figure S3 shows that the emissivity of the HDPE/VIS microfibrous membranes achieved a value of 0.88. The outstanding emissivity of the sample is primarily attributed to its dense three-dimensional network structure, increasing the scattering of sunlight and enhancing the thermal radiation capability. Figure 8a,b illustrates the reflectivity of the samples with respect to *M*_HDPEfs_ and *Pw*, respectively. The reflectivity curves show that the reflectivity increased from approximately 0.95 to 0.96 as *M*_*HDPEfs*_ increased from 37.1 to 94.2 g/m^2^. Similarly, the reflectivity increased from approximately 0.91 to 0.95 as *Pw* increased from 70 to 150 bar. These findings suggest that both higher *M*_*HDPEfs*_ and *Pw* contribute to enhanced reflectivity, which may be beneficial for improving the daytime radiative cooling performance.^37^ Figure 8c,d shows temperature–time curves for samples with different *M*_HDPEfs_ and *Pw*, respectively, during radiative cooling tests performed in Zhengzhou City, China (longitude: 113°41′26.480″E; latitude: 34°35′8.934″N) on May 29, 2024. Corresponding solar irradiance–time curves, which indicate fluctuations in solar irradiance during the test period, are presented in Figure 8e,f. The results show that higher *M*_*HDPEfs*_ and *Pw* values correlated with increased cooling rates and lower steady-state temperatures. For instance, the temperature for a sample with *M*_*HDPEfs*_ = 37.1 g/m^2^ was 8.4 °C, whereas a sample with 94.2 g/m^2^ exhibited a temperature of 12.9 °C. Similarly, a sample with *Pw* = 70 bar indicated a temperature of 5.8 °C, whereas a sample with *Pw* = 150 bar indicated a temperature of 8.3 °C. The daytime radiative cooling properties of the HDPE/VIS microfibrous membranes were further assessed through a series of experiments using a small cabin model exposed to solar radiation. Figure 8g,h combines the temperature–time curves and the infrared images, respectively. During the initial exposure to solar radiation, the temperature of all cabins increased; however, the rate of temperature increase differed significantly among the three conditions. After 15 min, the cabin covered with the HDPE/VIS microfibrous membrane reached a temperature of 56.9 °C, which was 3.5 °C lower than that of a cabin covered with cotton fabric (60.4 °C) and 5.2 °C lower than that of an uncovered cabin (62.1 °C). This significant difference in temperature highlights the superior thermal regulation and radiative cooling performance of the HDPE/VIS membrane, which effectively reduces heat absorption compared with conventional materials.

**Figure 8:**
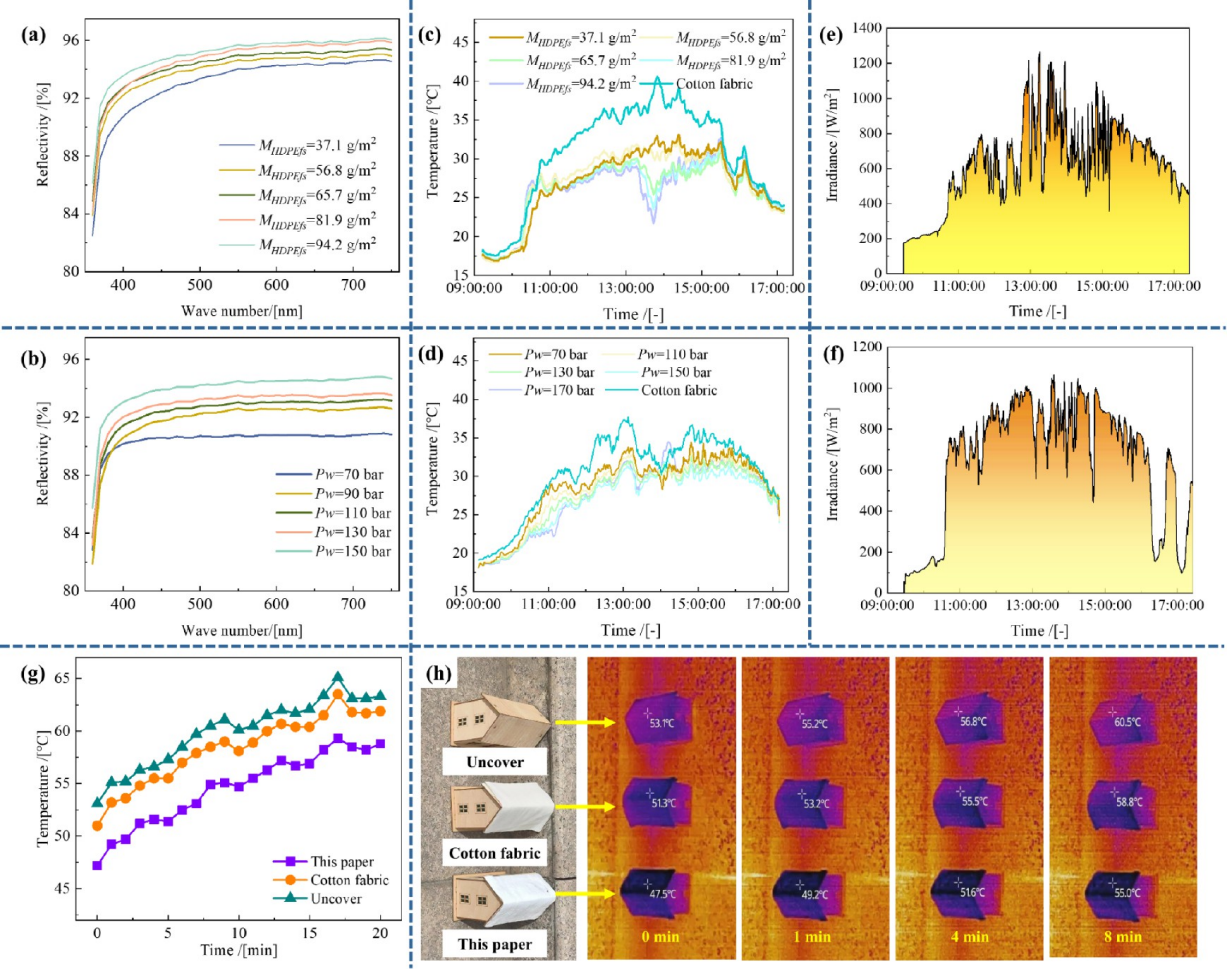
Reflectivity of prepared HDPE/VIS microfibrous membrane samples with different (**a**) *M*_*HDPEfs*_ and (**b**) *Pw*. Recorded temperature–time curves and corresponding solar irradiance–time curves of samples with different (**c**,**e**) *M*_*HDPEfs*_ and (**d**,**f**) *Pw*, during radiative cooling performance testing at Zhengzhou, China (May 29, 2024, 113°41′26.480″E, 34°35′8.934″N). (**g**) Temperature–time curves and infrared images (**h**) of the uncovered cabin and cabins covered separately by HDPE/VIS microfibrous membrane samples and cotton.

### 3.6 Softness Performance

Softness is an important comfort characteristic for the application of O-PPE. In this study, based on the principle of the Handle-O-Meter^38^ (H-O-M), a self-assembled H-O-M softness testing device was built to obtain the force–displacement curves of the fabric in MD and CD. As shown in Figure 9a and Video S5, when the sample was placed on the test base, the test blade contacted and compressed it, causing fiber deformation and displacement. This stage was referred to as the compression work (*Wa*). As the test blade continued to move downward, the sample came into contact with the edges of the test slot and began to bend, resulting in a bending deformation. This stage corresponded to bending work (*Wb*). With further displacement of the test blade, internal stress within the fibers developed to resist the external force, reaching a peak value (*P*). This stage represents the tensile work (*Wc*). Following the peak, some internal stress remained and continued to resist deformation, which was defined as residual stress work (*Wd*). Eventually, as the internal stress was exhausted, the sample bent and hung on both sides of the test slot, nearly perpendicular to it. The frictional work during this stage was denoted as *We*. The softness of the sample was evaluated by applying a vertical pressure. A higher applied force indicated a lower softness, meaning the material was more resistant to deformation. Furthermore, to quantitatively and comprehensively represent the softness performance of the samples, a numerical formula for evaluating overall softness was established using principal component analysis, based on the H-O-M method.^39^

1 $$St={A_{1}}^{*}Wa+{A_{2}}^{*}Wb+{A_{3}}^{*}Wc+{A_{4}}^{*}Wd+{A_{5}}^{*}We+{A_{6}}^{*}P$$

2 $$S=100-{\left[\left(St-Sc\right)/Sc\right]}^{*}100$$

**Figure 9:**
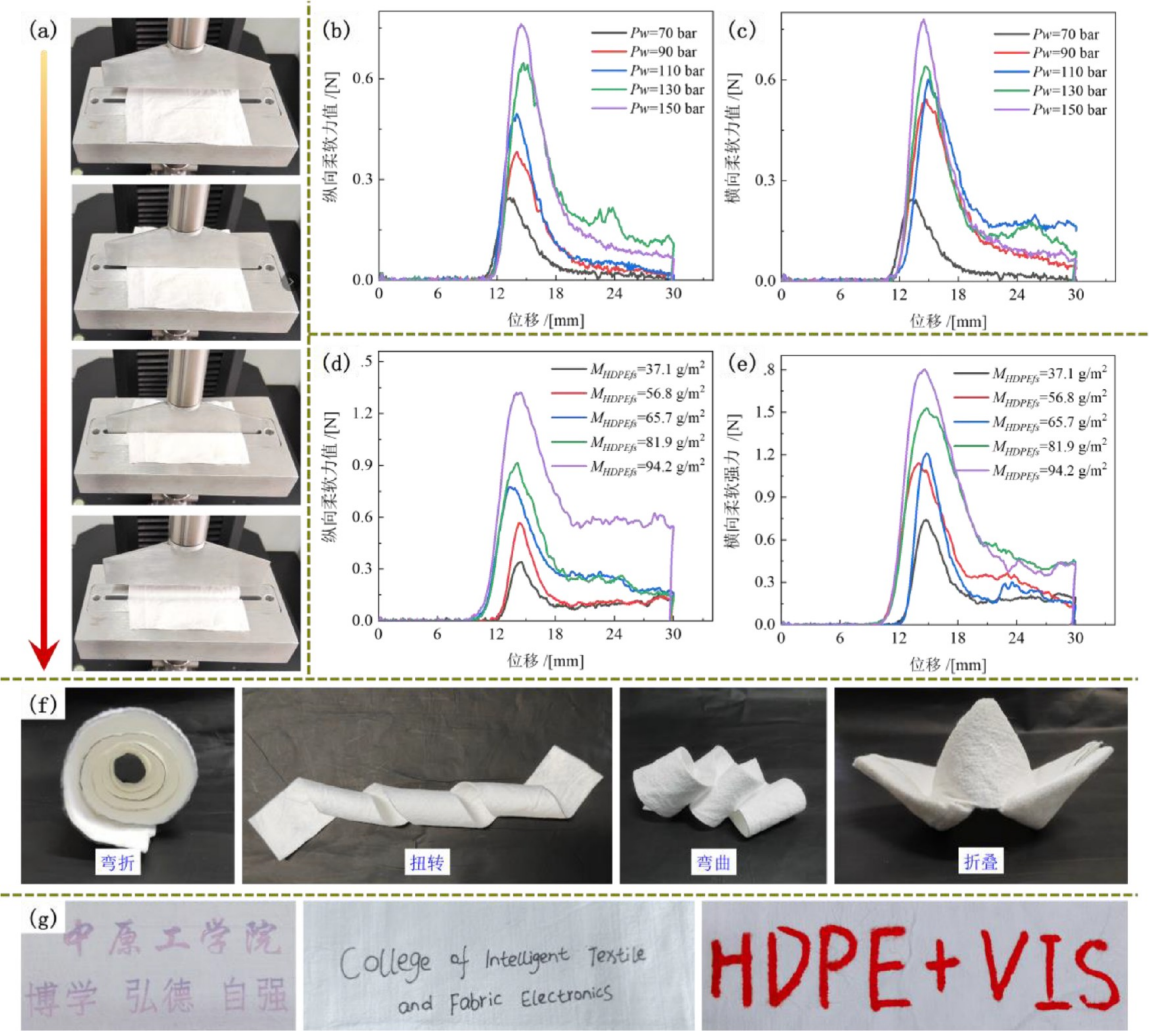
(**a**) Softness score testing process based on the H-O-M method. Softness force–displacement curves of HDPE/VIS microfibrous membranes prepared at different *Pw* values (**b**-MD, **c**-CD) and *M*_*HDPEfs*_ values (**d**-MD, **e**-CD). (**f**) Images of softness performance, i.e., folding, twining, bending, and folding, of HDPE/VIS microfibrous membrane samples. (**g**) Images of printing (portable mobile color printer, Zhuhai Yinwei Electronic Technology Co., Ltd., China) and writing performed using a marker pen and brush pen, respectively, on the HDPE layer.

Where *St* is the overall softness value, *S* is the softness score, and *A*_1_, *A*_2_, *A*_3_, *A*_4_, and *A*_5_ are the weight coefficients, which are 0.14, 0.19, 0.17, 0.17, 0.16, and 0.17, respectively, in the text. *Sc* represents the overall softness value of the standard sample.

Figure 9b,c shows the force–displacement curves for softness in the MD and CD of the HDPE/VIS microfibrous membrane samples prepared as different *Pw*, and the softness score curves are displayed in Figure S4. As *Pw* increased from 70 to 150 bar, the maximum soft-force value increased from 0.24 to 0.76 N in the MD, softness score decreased from 89.38 to 81.19, and the maximum soft-force value from 0.25 to 0.78 N in the CD, and the softness score decreased from 89.22 to 81.94. Typically, a higher *Pw* results in a denser fiber entanglement, which increases rigidity and reduces softness.^40^ Additionally, Figure 9d,e indicates that as *M*_*HDPEfs*_ increased to 94.2 g/m^2^, the maximum value increased to 1.32 and 1.80 N in the MD and CD, respectively, and the softness score decreased from 73.48 and 72.96. This shows that although increasing *M*_HDPEfs_ can enhance the mechanical strength, it reduces the softness. Figure 9f demonstrates the shape retention, flexibility, and deformation resistance of HDPE/VIS microfibrous membrane samples during winding, twisting, bending, and folding. The membrane can form sharp bends and folds without cracking or breaking, which is essential for the durability of the O-PPE in complex environments and under physical impact. Figure 9g shows the effects of printing and writing on the HDPE layer of the HDPE/VIS microfibrous membrane. The clear printing, smooth writing, and no smudging observed indicate the favorable ink absorption of the HDPE layer’s microfiber texture, thus rendering it effective for printing, labeling, and outdoor markings. Figure 10 compares a garment jacket fabricated by using the HDPE/VIS microfibrous membrane to conventional cotton and polyester counterparts. This cooling effect was particularly evident during physical activity, which indicates that the HDPE/VIS microfibrous membrane can effectively reduce heat buildup in the body during exercise, thereby enhancing comfort and athletic performance.

**Figure 10:**
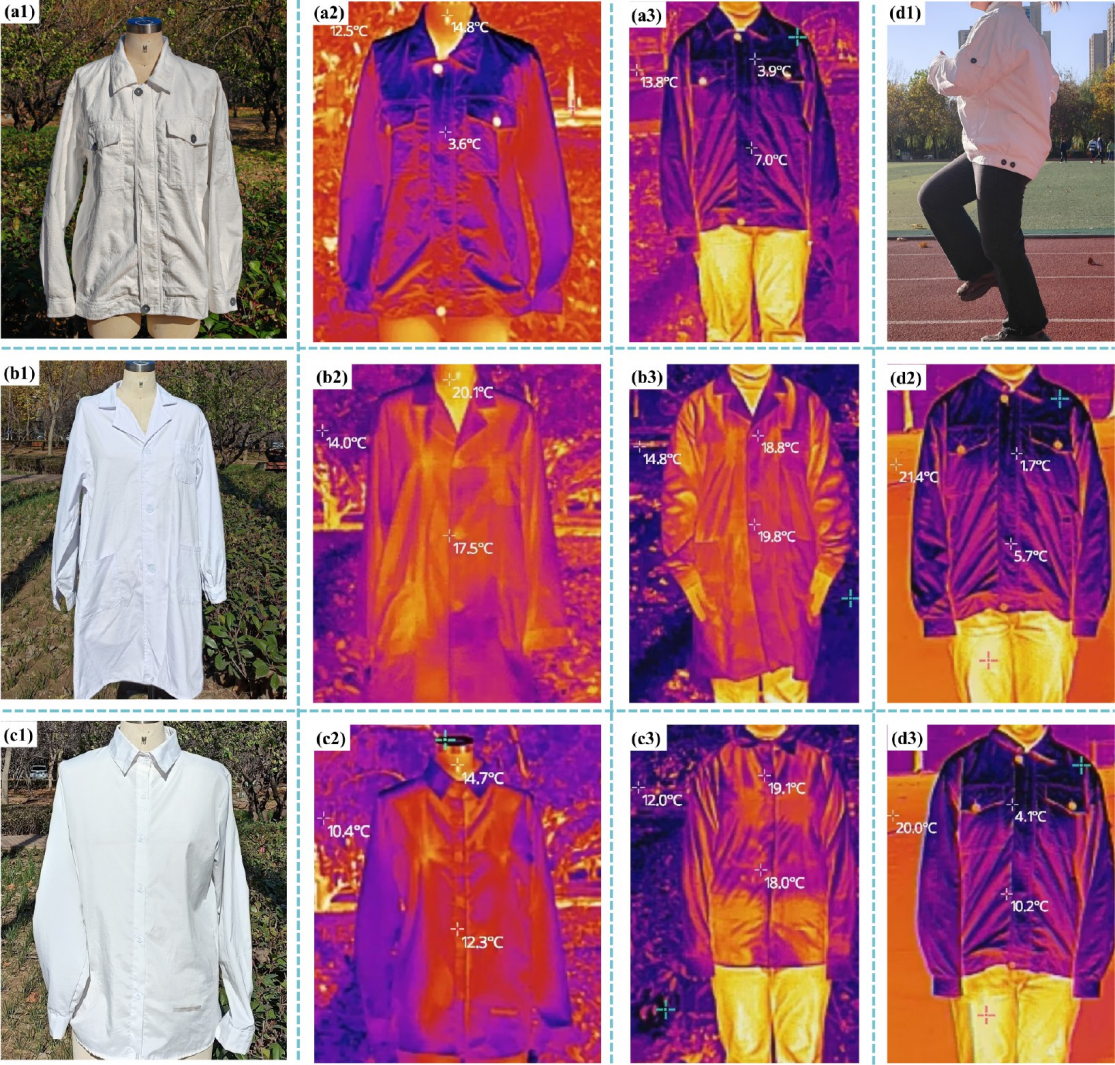
HDPE/VIS microfibrous membrane jacket (**a1**-physical image; **a2**-infrared image of a model wearing the jacket; **a3**-infrared image of a human body wearing the jacket). Cotton laboratory coat (**b1**-physical image; **b2**-infrared image of a model wearing the laboratory coat; **b3**-infrared image of a human body wearing the laboratory coat). Polyester shirt (**c1**-physical image; **c2**-infrared image of a model wearing the polyester shirt; **c3**-infrared image of a human body wearing the polyester shirt). HDPE/VIS microfibrous membrane jacket (**d1**-human body wearing a sports garment; **d2**-nfrared image before exercise; **d3**-infrared image after running 1 km).

## 4 Conclusions

In summary, an HDPE/VIS microfibrous membrane with a semiembedded structure was successfully prepared by integrating HDPE microfibrous webs with VIS fibrous webs via the hydroentanglement process for O-PPE applications. The HDPE microfibers, with a uniform fiber diameter ranging from 4 to 6 μm, partially penetrated the VIS layer, thus forming a tightly interlocked semiembedded bilayer membrane. The modal pore size and porosity of the HDPE/VIS microfibrous membrane decreased to 7.9 μm and 84.1%, respectively, as *Pw* increased to 150 bar, thus, resulting in a denser fibrous structure. This semiembedded structure, which comprised a VIS layer with rapid wettability and a hydrophobic HDPE layer, improved the liquid-management capacity. The *AOTI* improved significantly to 1036% when liquid water was applied to the VIS layer, whereas it decreased to −1377% when liquid water was applied to the HDPE layer. By adjusting the *M*_*HDPEfs*_ from 37.1 to 94.2 g/m^2^, the air permeability of the prepared samples decreased from 39.46 to 22.15 mm/s, whereas the *WVTR* remained above 1823.7 g/m^2^ ·24 h. Additionally, the samples exhibited superior tensile, bursting, and tearing strengths, which are crucial for ensuring durability and protection in harsh outdoor environments. Furthermore, the HDPE/VIS microfibrous membrane demonstrated a strong daytime radiative cooling performance. Specifically, it maintained surface temperatures 5.2 °C lower than those of uncovered surfaces owing to increased reflectivity as *Pw* increased from 70 to 150 bar. Overall, the HDPE/VIS microfibrous membrane effectively balances protection, comfort, and durability, thus rendering it a promising candidate that protects health and safety in advanced O-PPE applications.

## Supplementary Material













## Data Availability

The data underlying this study are not publicly available because they form part of a pending patent application and are therefore subject to confidentiality restrictions. The data are available from the corresponding author upon reasonable request and after the execution of a non-disclosure agreement.

## Notes

It encompasses the contact angles of water, cola, milk and coffee (Figure S1); the strain–stress, Young’s modulus and yield stress curves of HDPE/VIS microfibrous membrane samples (Figure S2); the emissivity curves of samples fabricated with *Pw* of 130 bar (Figure S3); the softness score curves (Figure S4); the main specifications of the HDPE pellets (Table S1) and VIS fibers (Table S2), and main hydroentanglement process paraments (Table S3) (PDF)
Movie S1, Movie S2: The AOTI test for HDPE layer and VIS layer, respectively (MP4)
Movie S3, Movie S4: The tearing process in CD and MD, respectively (MP4)
Movie S5: The softness process for sample (MP4)
MP4
MP4
