# Supplementary material for: Semiembedded HDPE/VIS Composite Membranes with Tailored Wettability and Superior Mechanical Durability for Outdoor Protective Applications
Source: ACS Omega. 2025 Jul 29;10(31):34857–70. doi: 10.1021/acsomega.5c03862 (PMC12355317; doi:10.1021/acsomega.5c03862)
Supplement: Supplementary file 1 [file ao5c03862_si_001.pdf]

Supporting information

Semi-embedded HDPE/VIS composite membranes with tailored wettability and superior mechanical durability for outdoor protective applications

Heng Zhang [1*], Qian Zhai[1], Qi Zhen[2], Hongbin lv[3], Kongmeng Ye[4]

1. College of intelligent textile and fabric electronics, Zhongyuan University of Technology, No. 1 Huaihe Road, Xinzheng County, 451191, Zhengzhou, Henan Prov., P.R. China
2. College of fashion technology, Zhongyuan University of Technology, No. 1 Huaihe Road, Xinzheng County, 451191, Zhengzhou, Henan Prov., P.R. China
3. ZFJ Textile machinery Co., LTD., No.258 Wutong Street, 450000, Zhengzhou, Henan Prov., P.R. China
4. Kingwills Advanced Materials Co., Ltd., No.166 Jianghai Road, Chongchuan, County, 226017, Nantong, Jiangsu Prov., P.R. China.

*** Corresponding Author**

Heng Zhang, Ph.D.,

E-mail: m-esp@163.com, zhangheng2699@zut.edu.cn.Authors

Tel: +86-156 3902 5712.

Address: Zhongyuan University of Technology, No.1 Huaihe road, Xinzheng, Zhengzhou city, Henan province, China

Figures

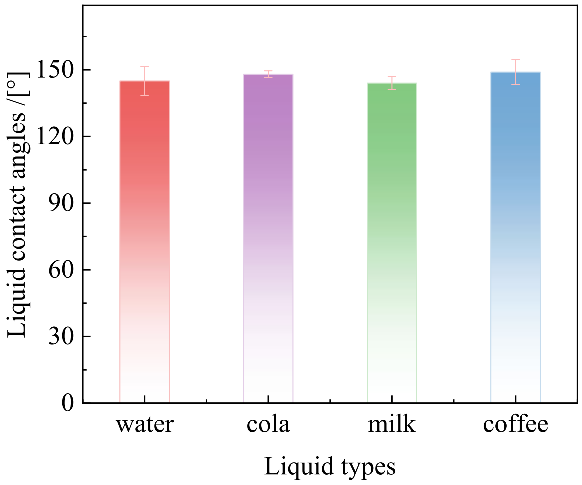

Figure S1 The contact angles of water, cola, milk and coffee.

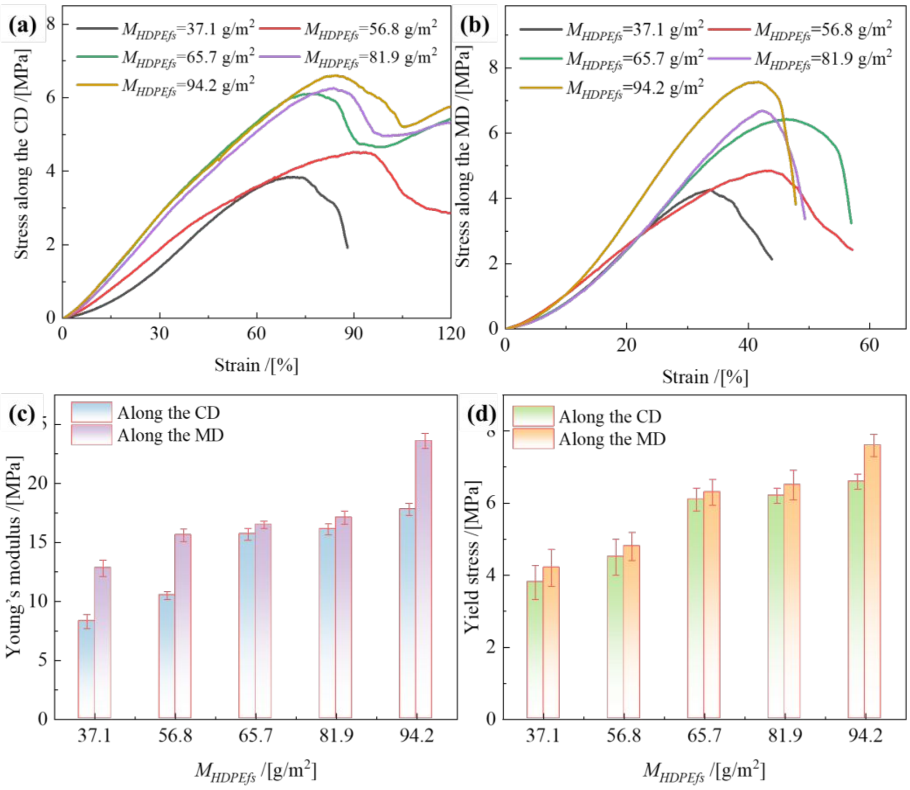

Figure S2 The strain-stress curves (**a**-CD, **b**-MD), young's modulus (**c**) and yield stress (**d**) of HDPE/VIS microfibrous membrane samples with different M_{HDPEfS} prepared at Pw = 110 bar.

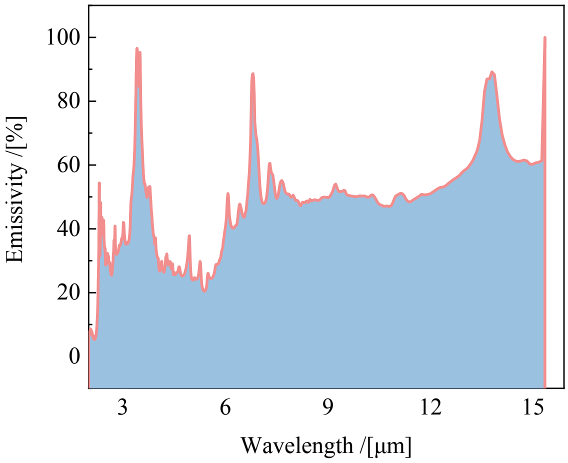

Figure S3 The emissivity curves of HDPE/VIS microfibrous membrane samples fabricated with *Pw* of 130 bar.

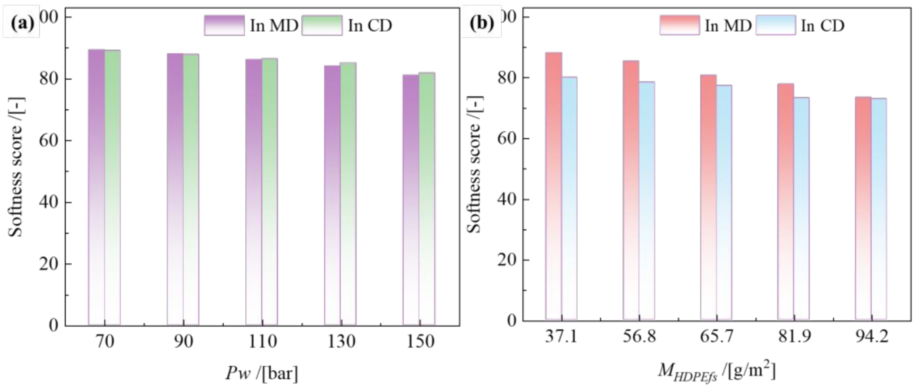

Figure S4 The softness score curves of HDPE/VIS microfibrous membrane samples with different (**a**) *Pw* and (**b**) M_{HDPEfS} prepared = 110 bar.

Tables

Tables S1. Main specifications of the HDPE pellets

Physical property	Testing standards	Data	Unit
Density	GB/T 1033	0.95-0.96	g/cm^3
Melt flow index	GB/T 3682	0.3-0.5	g/10min
Ash content	GB/T 9345	≤0.02	%

Tables S2. Main specifications of the viscose fibers

Physical property	Data	Unit
Linear density	1.56	dtex
Cut length	38	mm
Dry strength	≥2.10	cN/dtex
Wet strength	≥1.10	cN/dtex
Dry elongation	17-21	%
Whiteness	88	
Residual sulfur	≤0.008	%

Tables S3. Main hydroentanglement process paraments

Machine	Paraments	Setting	Unit
Hydroentanglement drive	flat screen	3	m/min
	Circular screen 1	3.11	m/min
	Circular screen 2	3.02	m/min
Pump	Clean water pump	2.5	bar
	Sewage pump	35	bar
	Multi-stage pump	5	bar
	High-pressure pump	70-150	bar
High pressure fan		40-100	Hz

Tables S4. Mechanical properties of the samples

M_{HDPEFS} /[g/m^2]	Young's modulus		Yield stress		Tensile break				Tear break		Bursting
	MD	CD	MD	CD	MD		CD		MD	CD	
	Stress /[MPa]	Stress /[MPa]	Stress /[MPa]	Stress /[MPa]	Force /[N]	Elongation /[%]	Force /[N]	Elongation /[%]	Force /[N]	Force /[N]	Force /[N]
37.1	12.8	8.3	4.2	3.8	139.9	58.2	126.5	77.4	66.6	52.8	187.4
56.8	15.6	10.5	4.8	4.5	159.6	40.5	149.6	72.9	81.6	60.8	231.2
65.7	16.5	15.7	6.3	6.1	211.8	40.9	183.2	72.1	100.1	86.2	288.8
81.9	17.1	16.1	6.5	6.2	220.3	40.1	206.2	66.3	110.5	95.5	303.4
94.2	23.6	17.8	7.6	6.6	249.6	36.9	217.7	57.3	127.4	104.7	364.1

note: MD = machine direction, CD = cross direction.
